# Micro-Clotting of Platelet-Rich Plasma Upon Loading in Hydrogel Microspheres Leads to Prolonged Protein Release and Slower Microsphere Degradation

**DOI:** 10.3390/polym12081712

**Published:** 2020-07-30

**Authors:** Miran Hannah Choi, Alexandra Blanco, Samuel Stealey, Xin Duan, Natasha Case, Scott Allen Sell, Muhammad Farooq Rai, Silviya Petrova Zustiak

**Affiliations:** 1 Program of Biomedical Engineering, School of Engineering, Saint Louis University, Saint Louis, MO 63103, USA; miranc2@illinois.edu (M.H.C.); ablanco3@illinois.edu (A.B.); samuel.stealey@slu.edu (S.S.); natasha.case@slu.edu (N.C.); scott.sell@slu.edu (S.A.S.); 2 Department of Orthopedic Surgery, Washington University in St. Louis, School of Medicine, Saint Louis, MO 63110, USA; duan.x@wustl.edu (X.D.); rai.m@wustl.edu (M.F.R.); 3 Department of Cell Biology & Physiology, Washington University in St. Louis, School of Medicine, Saint Louis, MO 63110, USA

**Keywords:** PRP therapy, microspheres, polyethylene glycol, drug delivery, injectable

## Abstract

Platelet-rich plasma (PRP) is an autologous blood product that contains a variety of growth factors (GFs) that are released upon platelet activation. Despite some therapeutic potential of PRP in vitro, in vivo data are not convincing. Bolus injection of PRP is cleared rapidly from the body diminishing its therapeutic efficacy. This highlights a need for a delivery vehicle for a sustained release of PRP to improve its therapeutic effect. In this study, we used microfluidics to fabricate biodegradable PRP-loaded polyethylene glycol (PEG) microspheres. PRP was incorporated into the microspheres as a lyophilized PRP powder either as is (powder PRP) or first solubilized and pre-clotted to remove clots (liquid PRP). A high PRP loading of 10% *w*/*v* was achieved for both PRP preparations. We characterized the properties of the resulting PRP-loaded PEG microspheres including swelling, modulus, degradation, and protein release as a function of PRP loading and preparation. Overall, loading powder PRP into the PEG microspheres significantly affected the properties of microspheres, with the most pronounced effect noted in degradation. We further determined that microsphere degradation in the presence of powder PRP was affected by platelet aggregation and clotting. Platelet aggregation did not prevent but prolonged sustained PRP release from the microspheres. The delivery system developed and characterized herein could be useful for the loading and releasing of PRP to promote tissue regeneration and wound healing or to suppress tissue degeneration in osteoarthritis, and intervertebral disc degeneration.

## 1. Introduction

Platelet-rich plasma (PRP) is an autologous preparation that concentrates platelets in a small volume of plasma through centrifugation [1,2]. Platelets contain a variety of growth and coagulation factors, adhesion molecules, cytokines/chemokines, and integrins [2,3]. Upon degranulation, the platelets release various growth factors (GFs) such as transforming growth factor-ß (TGF-β), platelet-derived growth factor (PDGF), insulin-like growth factor (IGF), basic fibroblast growth factor (FGF), vascular endothelial growth factor (VEGF), and epidermal growth factor (EGF) [4,5,6]. We and others have shown that the in vitro application of PRP promotes beneficial effects in various cells including dermal fibroblasts (implications in wound healing) and chondrocytes (implications in bone and cartilage regeneration) [7,8,9]. However, in vivo PRP therapy is less promising for a variety of reasons including, but not limited to, preparation methods, donor heterogeneity, and rapid clearance from the site of interest [5,10,11]. 

Donor variability can potentially be overcome through pooling PRP from multiple donors along with improved PRP preparation standards [12,13]. Rapid clearance from the injection site could be overcome by PRP encapsulation in a sustained-release device, such as a hydrogel. One simple approach would be to take advantage of the natural presence of fibrinogen in PRP [6], which, through thrombin activation, would result in a hydrogel for sustained release of GFs present in PRP. Several groups have attempted this release approach with improved results over a bolus PRP dose [14], but with disadvantages such as high burst release and the inability to control fibrin gel degradation. It is also important to note that PRP can contain a wide range of molecule concentrations, which also can affect fibrin gel formation [1,5]. In another approach, alginate hydrogel carriers have been used, where PRP GFs were able to bind to the ionotropic alginate, modulating their release [9]. Chitosan hydrogel has also been shown to prolong the release of PRP GFs up to 20 days, while preserving their bioactivity [15]. PRP has also been loaded in gelatin hydrogels (gelatin has natural affinity for various GFs), where sustained release from the gels has led to augmented bone regeneration [16] and wound healing [17]. Importantly, it was shown that bone regeneration (in a bone defect of rabbit ulna) was achieved only when PRP was incorporated in the gelatin hydrogel, but not with the PRP-free hydrogel or PRP-loaded fibrin gel, stressing the importance of prolonged and controlled PRP release [16].

For most therapeutic applications in the clinic, PRP is typically injected locally [2]. A site-specific injection of PRP has shown promise in the treatment of early knee osteoarthritis [11,18], improved healing of chronic ulcers [19], and regeneration of degenerated intervertebral discs [20]. It is, therefore, desirable that a PRP delivery device be implantable through a minimally invasive local injection. Several studies have focused on using injectable hydrogel microspheres [20,21] or injectable hydrogel formulations [22,23] for therapy purpose and have shown tremendous clinical potential. In this study, we also chose injectable microspheres to better control device shape and size as well as PRP pre-clotting, both of which would be important in an in vivo application.

An important feature of a minimally invasive delivery device is degradability, such that no device retrieval surgery is necessary while enabling repeat treatments. For that reason, most hydrogel formulations developed for PRP delivery are degradable, including the simple fibrin clot. However, for most currently developed delivery devices, precise control of degradation is not attainable as they are made of natural hydrogels such as fibrin, alginate, chitosan, and gelatin [9,15,21,24]. These hydrogels are degradable by enzymes (e.g., chitosan) or affected by the ionic composition of the environment (e.g., alginate), therefore their degradation would be dependent on enzyme concentration or ionic composition at the injection site. Here we chose to use a synthetic hydrogel, namely polyethylene glycol (PEG), which is biocompatible, inert to resist protein adsorption (important for release devices), and hydrolytically degradable with highly tunable degradation kinetics [25,26]. The added benefit of the chosen PEG formulation is the highly specific Michael-type addition crosslinking chemistry, which allows for easy protein encapsulation without compromising protein activity as shown by us previously [27,28].

The goal of this study was to utilize the tendency of PRP to clot to prepare interpenetrating network-like PEG hydrogel microspheres for prolonged microsphere degradation and release of PRP. We hypothesized that incorporating PRP in a nanoporous hydrogel network would cause the PRP to form micro-clots between crosslinks, which would displace water and reinforce the network. These micro-clots within a secondary network would sustain the release of PRP for an extended period with a reduced burst release compared to a polymer network with fully solubilized PRP components. Because of our choice of hydrogel microspheres for ease of delivery through a local injection, we were further concerned with burst release due to the higher surface to volume ratio of microspheres compared to a single bulk hydrogel. Lastly, we hypothesized that by displacing water and potentially forming a secondary network within the PEG, PRP would slow down the hydrolytic degradation of microspheres and change their physical and mechanical properties. 

## 2. Materials and Methods 

### 2.1. Materials

Four-arm PEG–acrylate (10 kDa; 4-arm PEG–Ac) and PEG–dithiol (3.4 kDa; PEG–diSH) were obtained from Laysan Bio (Arab, AL, USA). PEG-diester-dithiol-1 (3.4 kDa; PEGDD-1) was synthesized in-house as described by us previously [26]. Fluoro-max red microspheres (2.0 µm in diameter, ex./em. 542 nm/612 nm) and human lys-plasmin native protein were obtained from ThermoFisher (Waltham, MA, USA). FluoroSpheresTM carboxylate-modified, infrared, 0.1 µm in diameter (ex./em. 715 nm/755 nm) were obtained from Invitrogen (Carlsbad, CA, USA). Bovine serum albumin (BSA), lysozyme, 1X phosphate buffer solution (PBS), 1X Dulbecco’s phosphate buffer solution (DPBS), triethanolamine (TEA), Bradford Protein Dye Reagent, and Triton X-100 were obtained from Sigma Aldrich (St. Louis, MO, USA). Olive oil was obtained from Costco (Issaquah, WA, USA). A 60-mL syringe and needles were obtained from Becton Dickinson (Franklin Lakes, NJ, USA) and 100 µL gas-tight syringe was obtained from Hamilton Company (Reno, NV, USA). Tubing and T-junctions for the microfluidic setup were obtained from Valco Instruments (Houston, TX, USA).

### 2.2. Platelet-Rich Plasma Preparation

Pooled whole blood from 3 de-identified donors was obtained from Biological Specialty Company (Colmar, PA, USA) and prepared using the Harvest SmartPrep (Lakewood, CO, USA) processing kit following the manufacturer’s protocol. The concentrated platelets in the obtained PRP were lysed by a freeze-thaw cycle: −80 °C for 24 h, 37 °C for 1 h, and −80 °C for 24 h. To create a dry GF-rich powder, PRP was lyophilized (Lyophilizer, VirTis Sentry 2.0, Warminster, PA, USA) for 24 h and stored at −20 °C. 

**Figure 1:**
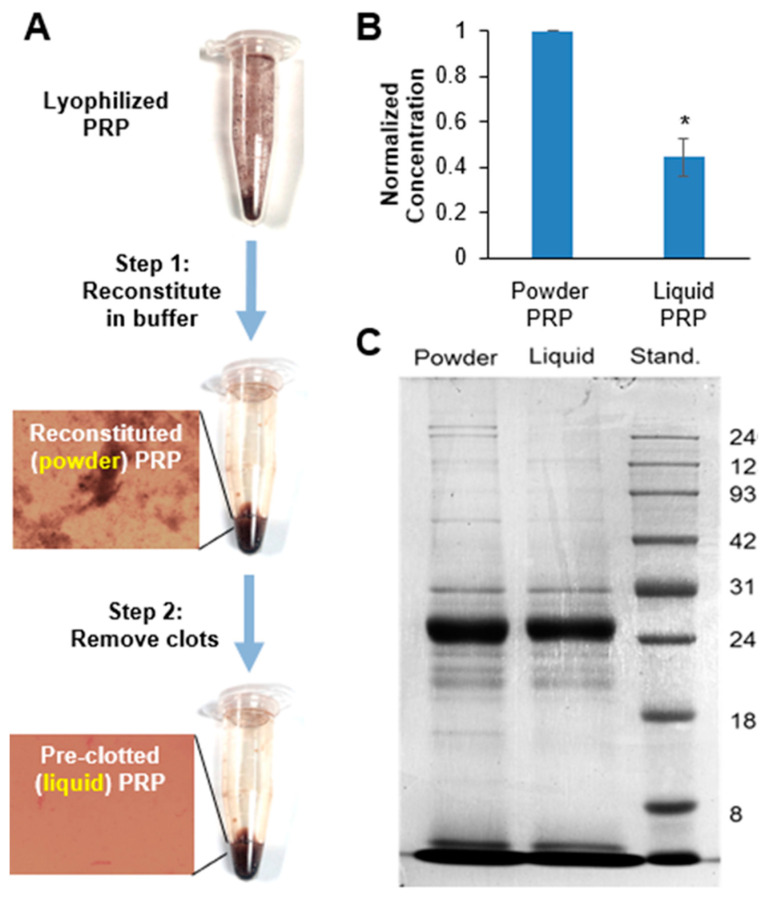
(**A**) Schematic of powder and liquid platelet-rich plasma (PRP) preparation. Lyophilized PRP is dissolved in buffer to prepare the powder PRP formulation. Powder PRP is then incubated for 24 h to form clots and clots are removed via centrifugation to prepare the liquid PRP formulation. Both powder and liquid PRP were used for further experiments. (**B**) Total protein content of reconstituted powder and pre-clotted liquid PRP. The total protein content was normalized by the protein content of the powder PRP. *denotes significant difference compared to powder PRP (*p* < 0.05). (**C**) Representative Coomassie-stained 1D electrophoresis gel.

Lyophilized PRP was used in microsphere fabrication in two preparations: by directly reconstituting it in a buffer and adding to the PEG hydrogel precursor solution (termed powder) or pre-clotted and clots removed prior to adding to the PEG hydrogel precursor solution (termed liquid) (Figure 1A). Note that because of the lysed platelets, even with clots removed, the liquid PRP is different from pure serum. There is minor clotting and activation that takes place upon hydrogel encapsulation, because of all the proteins released when platelets are lysed. To obtain the powder PRP preparation, lyophilized PRP was dissolved in TEA buffer (0.3 M in PBS, pH 7.4). To obtain the liquid PRP preparation, the reconstituted PRP solution was left on a shaker platform (Thermo Scientific 400110Q, Cole-Palmer, IL, USA) in a humidified incubator at 37 °C for 24 h to allow for clots to form. The clots were then removed by centrifugation for 3 min at 1500 RPM (IEC Centra CL2 Centrifuge, Thermo Electron Corporation, Milford, MA, USA) and decanting. Both PRP preparations were made fresh at 20% *w*/*v* and diluted to the desired final concentration in the hydrogel precursor solution as described below. The amount of PRP in both preparations was analyzed by running 1D sodium dodecyl sulfate-polyacrylamide gel electrophoresis (SDS-PAGE) following staining with colloidal Coomassie. Protein quantification was analyzed by Bradford protein dye reagent following the manufacturer’s instructions (Figure 1B,C).

### 2.3. PRP-Loaded PEG Microsphere Fabrication Via Microfluidics

**Figure 2:**
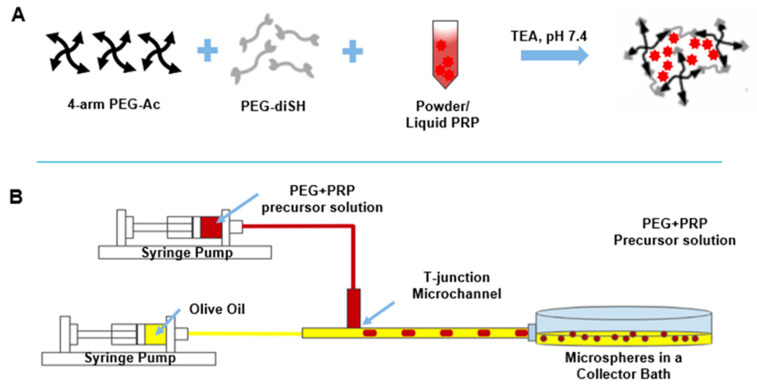
PRP-loaded microsphere fabrication via microfluidics. (**A**) Four-arm polyethylene glycol (PEG)–acrylate (PEG–Ac), PEG–dithiol crosslinker (PEG–diSH or PEG-diester-dithiol-1 (PEGDD-1)) -1) and either powder or liquid PRP were dissolved in 0.3 M triethanolamine (TEA) buffer of pH 7.4. A polymer network physically entrapping the PRP was then formed by Michael-type addition between the acrylate and thiol groups of the 4-arm PEG–Ac macromer and the dithiol crosslinker. (**B**) Olive oil and PRP-loaded PEG precursor solution were loaded into syringes and mounted on syringe pumps connected to a T-junction microchannel. PRP-loaded PEG microspheres were then collected in an olive oil bath and allowed to gel overnight.

All microspheres were loaded with 10% *w*/*v* PRP unless otherwise noted and were prepared with 10% *w*/*v* PEG unless otherwise noted. Briefly, PRP-loaded PEG microspheres were fabricated using 20% *w*/*v* stock solutions of 4-arm PEG–Ac and PEG–diSH or PEGDD-1 crosslinkers. Stock solutions were prepared in 0.3 M TEA of pH 7.4 for 4-arm PEG–Ac and PEG–diSH or in 0.3 M TEA pH 6.5 for PEGDD-1 (lower pH was used to allow for gelation kinetics similar to the PEG–diSH crosslinker). To prepare the hydrogel precursor solution, the 4-arm PEG–Ac and PEG–diSH or PEGDD-1 crosslinkers were combined in a 1:1 molar ratio of acrylate to thiol groups. Powder or liquid PRP were added directly to the precursor solution (Figure 2A) prior to gelation. Additionally, 1% *w*/*v* red fluorescent microbeads were added in certain experiments (e.g., degradation) to aid in microsphere visualization under fluorescence microscopy. When all components were added, the precursor solution was mixed by gentle pipetting and transferred into a 100-µL gas-tight glass syringe for microsphere fabrication. 

To fabricate hydrogel microspheres via microfluidics, the syringe loaded with the PRP-containing hydrogel precursor solution (disperse phase) was mounted on a programmable syringe pump (New Era Pump Systems, Farmingdale, NY, USA). A 60-mL plastic syringe containing olive oil (continuous phase) was mounted on a second programmable syringe pump (Figure 2B). Syringe pumps were programmed to dispense the olive oil at a flow rate of 1000 µL/min and the hydrogel precursor solution at a flow rate of 10 µL/min. Microfluidic tubing of 0.5 mm inner diameter was used for all experiments. Hydrogel droplets were collected in an olive oil bath and left overnight to form microspheres. Microspheres were then transferred to a centrifuge tube and centrifuged for 3 min at 1500 RPM (IEC Centra CL2 Centrifuge, Thermo Electron Corporation, Milford, MA, USA). Upon centrifugation, the oil was decanted and the microspheres were washed once with 1% *w*/*v* Triton X-100 followed by three washes in PBS. The oil bath and wash buffers were collected and analyzed for loading efficiency as described below. 

### 2.4. PRP Loading Efficiency

Collector oil bath and microsphere washing buffers were collected in a centrifuge tube as described above. The solutions were transferred into a separatory funnel and shaken vigorously to create a fine water-oil emulsion. The emulsion was left to separate overnight and the aqueous layer, which contained PRP proteins, was collected and stored at −80 °C or used immediately for analysis. Total protein content was analyzed by Bradford protein dye reagent following the manufacturer’s protocol.

### 2.5. Characterization of Microsphere Diameter and Degradation via Microscopy 

Microspheres were imaged using an inverted microscope (Axiovert 200, Zeiss, Germany) at 10× magnification. Microsphere images were used to characterize microsphere diameter, as has been done previously by us and others [28,29]. For imaging, microspheres suspended in either olive oil (pre-wash for initial diameters) or PBS (post-wash for swollen diameters) were mixed by gentle pipetting and 3 samples of 30 µL per batch (from 3 batches minimum) were taken out for analysis. Microsphere diameters were analyzed via ImageJ (free of charge from the NIH at https://imagej.nih.gov/ij/) using the Line Analyzer plug-in. Percent swelling was calculated as:

(1) $$\%\;swelling=\frac{Mean\;diameter_{swollen}-Mean\;diameter_{initial}}{Mean\;diameter_{initial}}\times 100$$

To analyze polydispersity of microspheres, percent coefficient of variance, %CV, was calculated as:

(2) $$\%CV=\left(\frac{Standard\;Deviation}{Mean}\right)*100\%$$

For degradation measurements, microspheres (loaded with red fluorescent beads for visualization) were collected and washed as described above. Microspheres (50 µL volume per sample) were incubated in 1X PBS (500 µL volume total) with shaking in a humidified incubator at 37 °C. At desired time points (1–30 days), 30 µL samples were taken and imaged as described above. Degradation was followed by an increase in the microsphere diameter and complete degradation was noted when more than 95% of the microspheres were degraded in the incubation medium. Microspheres were considered degraded when the red fluorescent beads were released in the incubation medium (as opposed to being confined in individual microspheres). 

### 2.6. Rheology

For rheological testing 200 µL volume of PRP-loaded and PEG-only microspheres were encapsulated within 200-µL volume bulk PEG hydrogels (for a total of 400 µL sample) made into 20 mm discs of 500 µm thickness. The microsphere-loaded gels were then swollen for 2 h in DI water and excess water was carefully blotted with a Kimwipe^TM^. The gels were placed on a rheometer between two 20 mm parallel plates (AR-2000ex rheometer, TA Instruments, New Castle, DE) and measured at 25 °C at a frequency of 1–10 rad/s and a constant 1% strain.

### 2.7. Characterization of PRP Release from PEG Microspheres

For release experiments, 150 µL of PRP-loaded microspheres were placed in microcentrifuge tubes filled with 1.5 mL of 1X PBS and incubated with shaking at 37 °C until degradation. At desired time points, 750 µL aliquots were collected and replenished with fresh PBS. Collected releasates were stored at −80 °C until analysis. Releasates were analyzed for total protein content by Bradford protein dye reagent following the manufacturer’s instructions as described by us previously [8]. Total protein content in aliquots collected at different time points (*M_i_*) was normalized by the total loaded protein (*M_inf_*). 

Using a modified form of Fick’s Law, the effective diffusion coefficient of PRP was calculated for short release times as [30]:

(3) MiMinf=6[Detπa2]12−3(Deta2)

where *D_e_* is the effective diffusion coefficient, *t* is the time, and *a* is the radius of the hydrogel microspheres (50 μm). 

### 2.8. In Vivo PRP-Loaded Microsphere Degradation 

The Washington University Animal Care and Use Committee approved this study. All mice were purchased from the Jackson laboratory (Bar Harbor, ME, USA) and housed in groups in individually ventilated cages (5 mice/cage). All animals had ad libitum access to low-fluorescence mouse chow and water and a 12:12 h light:dark cycle. In this experiment, 10-wk-old male C57BL/6J mice were used. Under anesthesia, the right knees were kept in a flexed position. Infrared FluoroSphere (715/755)-loaded PEG hydrogel microspheres (15 μL per joint) with or without PRP (n = 3) were injected intra-articularly on day 0 with a 30-gauge needle. The fluorescent signal was followed up to 42 days after injection by an In Vivo Imaging System (Xenogen In Vivo Imaging System 50, Perkin Elmer). Mice receiving PBS injection served as control.

### 2.9. Statistical Analysis 

All data are presented as mean values with error bars of ± one standard deviation, determined from minimum of three independent experiments with 3–6 samples each. When individual microspheres were analyzed, a total of 50–100 microspheres were analyzed per each data point. Multiple samples were compared using single factor analysis of variance (ANOVA) followed by Tukey’s post-hoc analysis. Two-tailed Student’s t-test was used to determine statistical significance between two groups (*p* < 0.05). 

## 3. Results

### 3.1. PRP-Loaded Microspheres Fabrication via Microfluidics 

We used both powder and liquid PRP, where the amount of PRP (10% *w*/*v* unless otherwise stated) was reported as the amount of original lyophilized powder used (which is the same for both powder and liquid preparations). However, due to the additional pre-clotting step, the amount of total protein in the liquid preparation was expected to be lower than that of the powder formulation (Figure 1A). As expected, liquid PRP had 2.23-fold less total protein content compared to powder PRP (Figure 1B,C). Qualitatively, we also noted that the powder PRP showed darker gel bands (higher amount) for the higher molecular weight proteins compared to liquid PRP. This finding was consistent with our expectation that larger molecules would be removed with the additional pre-clotting step. 

**Figure 3:**
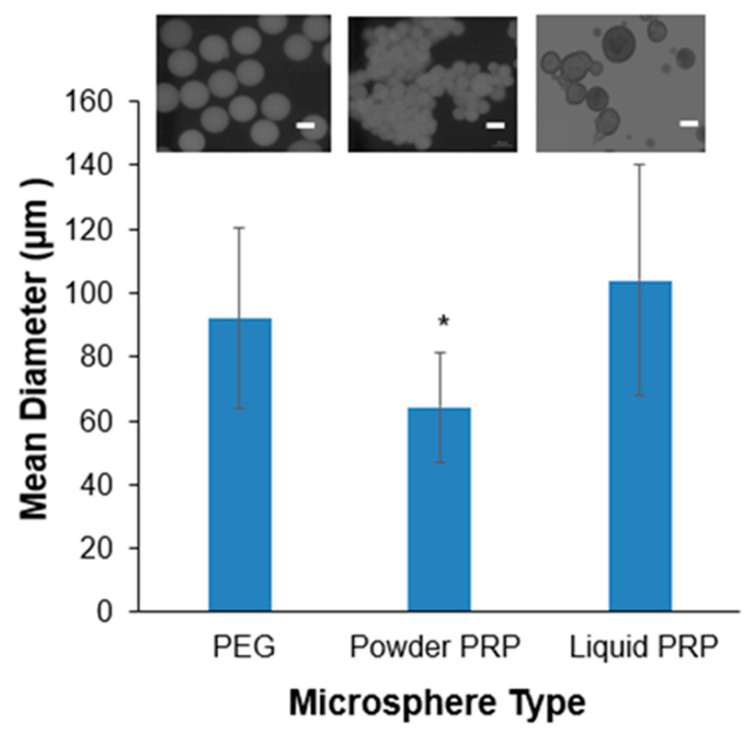
Mean diameters of PEG-only, powder PRP, and liquid PRP-loaded microspheres at olive oil flow rate of 1000 μL/min and PEG precursor solution flow rate of 10 μL/min. Microspheres were prepared with 4-arm PEG–Ac and PEG–diSH as a crosslinker at 10% *w*/*v* in PEG. Powder and liquid PRP were loaded at 10% *w*/*v*. All diameters were measured in oil, prior to microsphere washing. * indicates statistical difference from all other conditions (n = 100; *p* < 0.05). Inset: Representative microscopy images of microspheres. Scale bar is 100 µm.

We next fabricated PRP-loaded PEG hydrogel microspheres via microfluidics as shown in Figure 2. Flow rates of 1000 µL/min for the continuous oil phase and 10 µL/min for the disperse PEG phase were used for all experiments, resulting in microspheres of ~100 µm in diameter. PRP, in either the powder or the liquid preparation, was loaded directly in the hydrogel precursor solution prior to microsphere fabrication. The PRP loading efficiency was ~99% for both powder and liquid PRP-loaded PEG microspheres as calculated by the amount of PRP lost in the collector oil bath and PBS washes. Loading powder or liquid PRP in the microspheres resulted in statistically significant differences in mean diameter between the two conditions, with the liquid PRP-loaded ones being larger (Figure 3). Loading powder PRP led to a significant 30% decrease in mean diameter compared to PEG-only microspheres and loading liquid PRP led to a slight but not significant 12% increase in mean diameter compared to PEG-only microspheres. 

### 3.2. Microspheres Swelling and Modulus as a Function of PRP Loading

**Figure 4:**
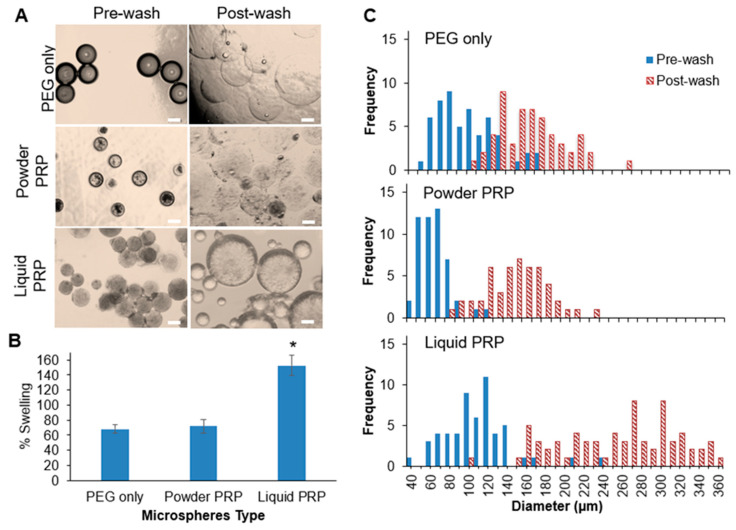
Swelling of PEG microspheres as a function of PRP loading. (**A**) Images of PEG-only, powder PRP, and liquid PRP-loaded microspheres before and after swelling. Scale bar is 100 µm. (**B**) Percent swelling of PEG-only, powder PRP, and liquid PRP-loaded microspheres * indicates statistical difference from all other conditions (n = 50; *p <* 0.05). (**C**) Representative histograms for the size distribution of one batch of fabricated microspheres of PEG-only, powder PRP, and liquid PRP. Microspheres were prepared with 4-arm PEG–Ac and PEG–diSH as a crosslinker at 10% *w*/*v* in PEG. Powder and liquid PRP were loaded at 10% *w*/*v*.

Swelling was determined by the increase in microsphere diameter upon soaking in a PBS buffer compared to the initial microsphere diameter upon fabrication (prior to washing). The loading of powder PRP had no effect on microsphere swelling, but the loading of liquid PRP led to a significant 2.25-fold increase in microsphere swelling compared to PEG-only microspheres (Figure 4A,B). All microspheres were also assessed for their size distribution before (pre-wash) and after washing and swelling (post-wash). As expected, for all microspheres (PEG-only, powder PRP-loaded and liquid PRP-loaded) we observed a shift towards higher diameters upon swelling (Figure 4C). We also noted a wider size distribution for liquid PRP-loaded microspheres compared to PEG-only and powder PRP-loaded ones, both pre- and post-wash. Calculated %CVs for PEG-only, powder PRP, and liquid PRP-loaded microspheres were 21.7%, 15.3%, and 27.8%, respectively.

**Figure 5:**
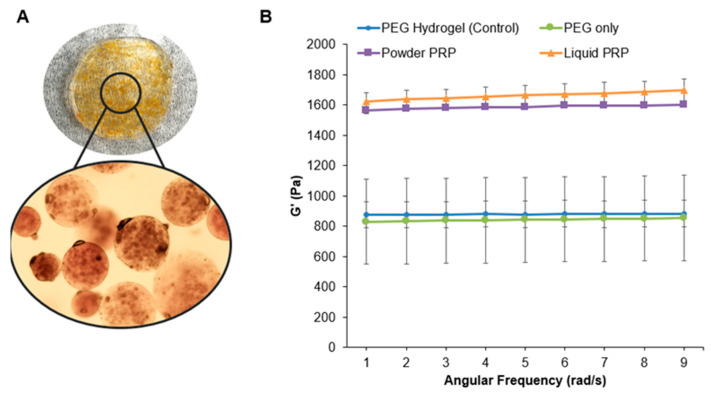
Storage moduli of microspheres as a function of PRP loading. (**A**) Representative images of powder PRP-loaded microspheres encapsulated in a bulk PEG hydrogel (10% *w*/*v* in PEG) for rheology measurements. (**B**) Representative data for storage moduli of microspheres as a function of PRP loading (n = 3). Microspheres were prepared with 4-arm PEG–Ac and PEG–diSH as a crosslinker at 10% *w*/*v* in PEG. Powder and liquid PRP were loaded at 10% *w*/*v*.

To determine the microspheres’ modulus via rheology, microspheres were physically trapped in a bulk PEG hydrogel as shown in Figure 5A. The modulus of the bulk hydrogel without trapped microspheres was measured as a control. The loading of both powder and liquid PRP led to a significant ~twofold increase in the storage modulus of microspheres compared to PEG-only microspheres (Figure 5B). There was no difference between the modulus of powder vs liquid PRP-loaded microspheres. As expected, the loading of PEG-only microspheres did not affect the modulus of the bulk PEG hydrogel. 

### 3.3. Microsphere Degradation in Vitro and in Vivo as a Function of PRP Loading

**Figure 6:**
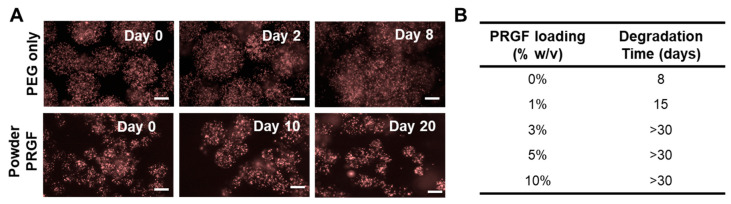
Degradation of microspheres as a function of PRP loading. (**A**) Microscopic images of 10% *w*/*v* powder PRP-loaded microspheres in PBS. Microspheres were loaded with 1% *w*/*v* red-fluorescent beads for visualization. Degradation was noted by fluorescent beads being dispersed in the surrounding medium as opposed to being confined in the microspheres. (**B**) Degradation time for powder-PRP loaded microspheres as a function of PRP concentration. Microspheres were prepared with 4-arm PEG–Ac and PEG–diSH as a crosslinker at 10% *w*/*v* in PEG.

For most of the degradation studies, we investigated powder PRP loaded microsphere compared to PEG-only microspheres due to our interest in micro-clotting (achievable only with powder PRP); degradation of liquid PRP-loaded microspheres is discussed later. The PEG polymers used here degrade by hydrolytic degradation due to a thioester bond formed upon the crosslinking of the acrylate in 4-arm PEG–Ac and the terminal thiol in the PEG-based crosslinker. To study the effect of PRP loading on degradation, PEG microspheres with and without powder PRP (10% *w*/*v*) were incubated in PBS and imaged at pre-determined time points (Figure 6A). Powder PRP-loaded microspheres had a significantly higher degradation time (>30 days) compared to PEG-only microspheres (8 days). Note that the degradation experiment for PRP-loaded microspheres was capped at 30 days (prior to complete degradation), even though some non-degraded microspheres were still observed at 2 months. Complete degradation was considered when >95% of microspheres were degraded (beads were released in the surrounding medium). We also determined that degradation was dependent on the concentration of loaded PRP (Figure 6B). Any amount of PRP loaded led to an increase in degradation time compared to PEG-only microspheres, but of differing magnitude. The loading of 1% *w*/*v* PRP led to ~twofold increase in degradation time. A loading of a 3% or higher PRP concentration led to a degradation time of >30 days. 

**Figure 7:**
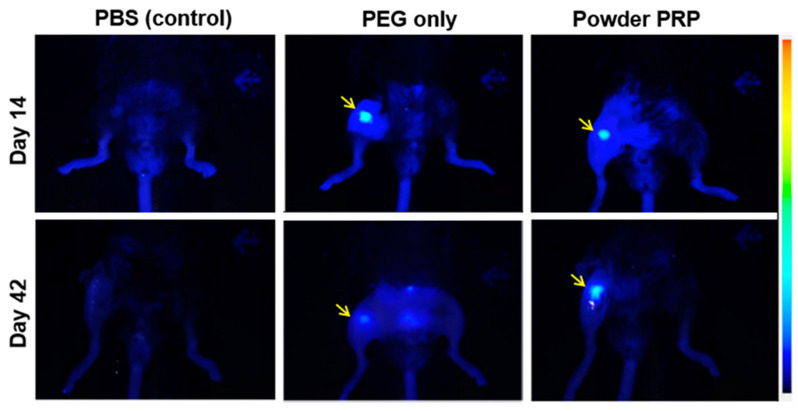
Degradation of PEG microspheres as a function of PRP loading in mouse knee. Microspheres were prepared with 4-arm PEG–Ac and PEG–diSH as a crosslinker at 10% *w*/*v* in PEG. Powder PRP was loaded at 10% *w*/*v*. Infrared FluoroSphere-loaded PEG hydrogel microspheres with or without PRP were injected into the mouse knee by intra-articular injection. The fluorescent signal was followed up to 42 days after injection by an In Vivo Imaging System. PBS injection served as a control. Results showed that both PEG-only and PRP-loaded microspheres were present in the joint space (indicated by yellow arrow) 14 days after injection but only PRP-loaded microspheres remained there until 42 days after injection.

To test in vivo degradation, we injected intra-articular PEG-only and powder PRP-loaded PEG microspheres (10% *w*/*v* in PRP) into the right knee joint space of healthy mouse models. The microspheres were rendered fluorescent by the incorporation of near infrared fluorescent microspheres (λ = 715/755). Corroborating in vitro data, we observed that PRP-loaded microspheres degraded slower than PEG-only microspheres (Figure 7). IVIS imaging showed that both PEG-only and PRP-loaded microspheres were present in the right knee joint space 14 days after injection, but only PRP-loaded microspheres remained there 42 days after injection.

### 3.4. Effect of PRP Micro-Clotting on PRP-Loaded PEG Microsphere Degradation

**Figure 8:**
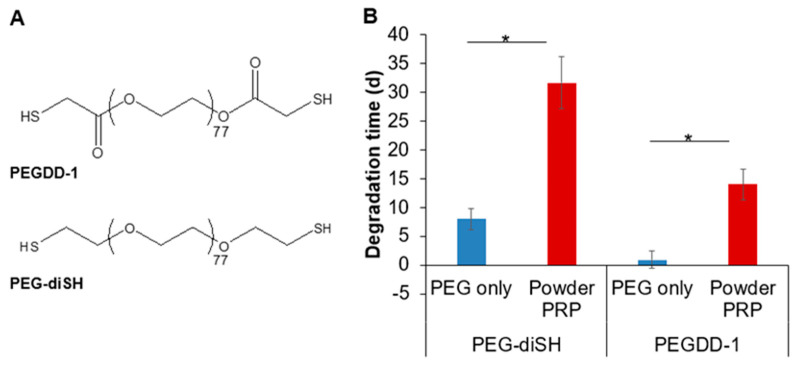
Degradation of PEG-only and PRP-loaded PEG microspheres in PBS as a function of the crosslinker used for microsphere preparation. (**A**) Chemical structures of PEGDD-1 and PEG–diSH. (**B**) Microsphere degradation time. Microspheres were prepared with 4-arm PEG–Ac and PEG–diSH or PEGDD-1 as a crosslinker at 7.5% *w*/*v* in PEG. Powder PRP was loaded at 10% *w*/*v*. * denotes significant difference between groups (n = 50; *p* < 0.05).

Here, we determined whether clotting was responsible for the significantly delayed degradation of PEG microspheres upon PRP loading. For all prior experiments PEG–diSH crosslinker was used to prepare hydrogel microspheres loaded with 10% *w*/*v* PRP; however loaded microspheres did not degrade completely even at 2 months (degradation experiments were capped at 30 days as noted earlier; Figure 6). To allow us to determine nuances in degradation times as a function of PRP clotting, we instead used a custom-synthesized fast-degrading crosslinker, namely PEGDD-1 [25,26] (Figure 8A). Note that both PEG–diSH and PEGDD-1 have the same molecular weight (3.4 kDa) and have almost identical chemical structure except for an ester moiety near the thiol end group for the PEGDD-1 crosslinker. PRP was loaded at the same 10% *w*/*v* for both PEG-based microspheres. We have previously compared hydrogels made with PEG–diSH and PEGDD-1 for their ability to sustain the release and preserve the stability of encapsulated proteins [31]. Overall, while both hydrogel types preserved protein stability, the diffusivity and release of model proteins was faster from gels made with PEGDD-1, due to its significantly faster degradation compared to gels made with PEG–diSH [31]. Here, we also decreased the total PEG concentration of the hydrogels from 10% to 7.5% *w*/*v* to further speed up degradation. Lowering the polymer concentration resulted in lower crosslink density, hence, a faster hydrolytic degradation. We confirmed that both unloaded and PRP-loaded microspheres made with the ester-containing crosslinker PEGDD-1 had faster degradation time compared to the non-degradable PEG–diSH crosslinker (Figure 8B). Importantly, for both crosslinkers, PRP-loading significantly increased degradation time. PRP-loaded microspheres made with PEGDD-1 degraded in ~2 weeks compared to PEG-only microspheres made with PEGDD-1, which degraded in ~1 day. 

Note that microspheres prepared with PEGDD-1 and loaded with liquid PRP degraded in ~5 days (Figure S1), which was faster than the powder PRP, but slower than PEG-only microspheres. Further, we loaded microspheres with a high concentration of 10% *w*/*v* of two other model proteins, namely BSA (66 kDa) and lysozyme (14 kDa) (Figure S2), which could serve as macromolecular fillers, displacing water molecules, but would not form clots. We observed that BSA encapsulation resulted in microspheres degradation time of ~8 days, which was higher than PEG-only microspheres but significantly lower than that of powder PRP. Encapsulation of lysozyme did not lead to changes in degradation time compared to PEG-only microspheres; lysozyme-loaded microspheres degraded in ~1 day. Collectively, this data indicates that simply displacing water from the PEG network could not explain the significantly slower degradation of powder PRP-loaded microspheres.

**Figure 9:**
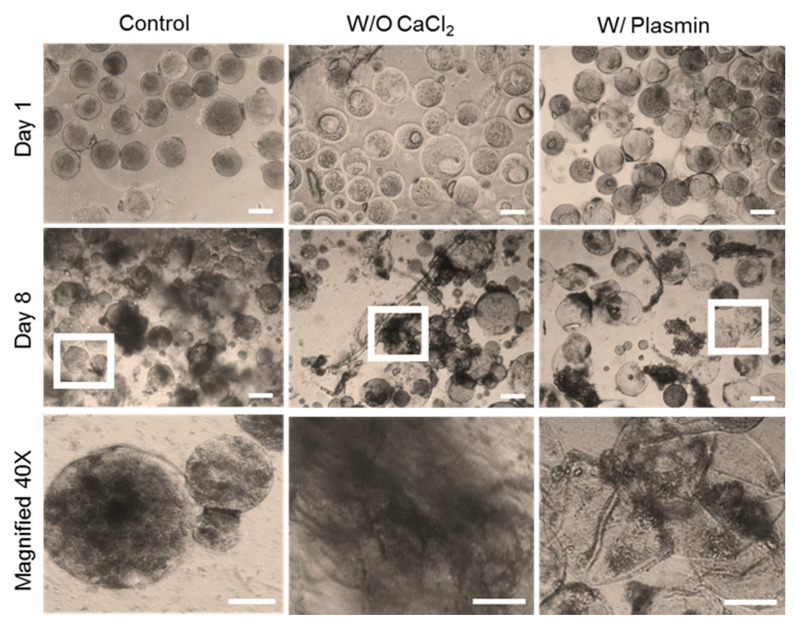
Degradation of PRP-loaded microspheres as a function of plasmin addition or absence of Ca^2+^ during fabrication. Representative microscopic images of microspheres were taken on day 1 and 8. Scale bar is 100 µm for day 1 and day 8; scale bar is 50 µm for magnified images, day 8. Microspheres were prepared with 4-arm PEG–Ac and PEGDD-1 as a crosslinker at 7.5% *w*/*v* in PEG. Powder PRP was loaded at 10% *w*/*v*.

We next tested whether preventing clot formation or braking up clots during fabrication would affect the PRP-loaded microsphere degradation time. Plasmin was used to break up clots upon formation as plasmin is the main enzyme of the fibrinolytic system that cleaves fibrin to soluble degradation products [32]. Ca^2+^-free buffer (DPBS) was used to prevent clotting as Ca^2+^ plays a critical role in the coagulation cascade and is used clinically to clot PRP [33]. First, the activity of plasmin was tested by dissolving 10% *w*/*v* PRP in TEA buffer with or without added plasmin (Figure S3). After 24 h of incubation, clotting was observed in the TEA only sample, but not when plasmin was present, indicating that plasmin was active and able to prevent clot formation at the chosen concentration of 1% *w*/*v*. We next used plasmin or Ca^2+^-free buffer during microsphere fabrication and observed their effect on powder PRP-loaded microsphere degradation compared to control microspheres (powder PRP-loaded microspheres soaked in PBS only) (Figure 9). Overall, for both Ca^2+^-free medium and plasmin conditions, we noted increased degradation and microsphere breakage at day 8 compared to the control condition. 

### 3.5. PRP Release from Liquid and Powder PRP-Loaded PEG Microspheres

**Figure 10:**
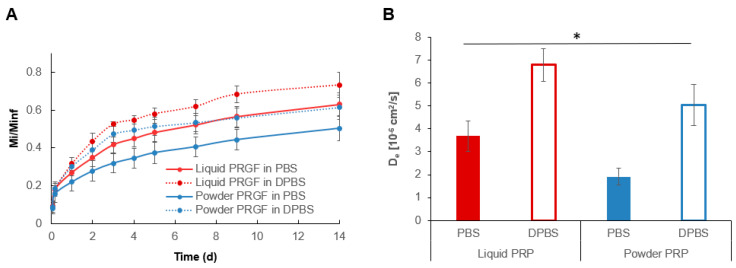
Release of powder and liquid PRP from PEG microspheres. (**A**) Normalized release profiles of powder PRP and liquid PRP-loaded microspheres. Both PBS and DPBS were used as release buffers where DPBS prevented clotting of PRP upon release. (**B**) Calculated effective diffusion coefficient for PRP release from powder PRP and liquid PRP-loaded microspheres in PBS and DPBS buffers. * denotes significant difference between all groups (n = 6; *p* < 0.05).

For release experiments, the total loaded amount of powder and liquid PRP (*M_inf_*) was determined by measuring the total protein content of precursor solutions using a Bradford assay. In addition to a PBS release buffer, DPBS (Ca^2+^-free) was used as a control to determine whether inhibiting clotting during release would affect PRP release. In PBS, PRP exhibited sustained release from PEG microspheres over 14 days, with an initial burst release where ~20% of the proteins were released in the first 24 h for both powder and liquid PRP (Figure 10A). A total of ~50% of the protein content was released in the first 7 days for liquid PRP and a total of ~40% protein content was released for powder PRP. At 14 days ~66% and ~50% of the total protein content was released for liquid and powder PRP, respectively. In DPBS (when clotting was inhibited) more PRP was released at all time points compared to PBS for both liquid and powder PRP-loaded microspheres. Further, *D_e_* was overall significantly higher for liquid than powder PRP-loaded microspheres in both PBS (1.93-fold) and DPBS (1.35-fold) (Figure 10B). *D_e_* was also higher in DPBS than PBS for both liquid PRP (1.84-fold) and powder PRP-loaded microspheres (2.64-fold), underscoring the effect of clotting on protein release.

## 4. Discussion

PRP has begun to emerge as a promising, low-cost, and minimally invasive therapy that delivers highly concentrated GFs and other bioactive molecules to damaged tissues, reducing pain and inflammation and contributing to tissue regeneration [2]. However, the challenge remains in the delivery method of PRP. For example, when PRP is delivered via intra-articular injections to treat knee osteoarthritis, secreted GFs and bioactive molecules are rapidly cleared from the injection site [4]. The lymphatic system removes proteins with a molecular weight >1 kDa from joints within just a few hours [34]. Additionally, bolus PRP injection shows a burst release (70%) of GFs upon platelet activation and almost 100% release within an hour [2]. Rapid clearance of GFs results in low therapeutic effects, an impediment which could be overcome by developing a sustained release device for PRP. The goal of this project was to develop and characterize delivery system consisting of injectable PRP-loaded PEG hydrogel microspheres. Further, we explored the possibility of using the natural clotting ability of PRP to influence both hydrogel degradation and PRP protein release. We anticipated that PRP clotting inside the PEG network will influence all other microsphere properties, including their sizes and mechanical strength. Hence, the nature of the clotting and its effect on microsphere properties and PRP release was thoroughly investigated. 

We have previously studied PRP release from a bulk PEG hydrogel and demonstrated that the sustained release had beneficial effects on primary chondrocytes, surpassing that of a bolus PRP dose [7]. Separately, we have also shown a large burst release of PRP with a majority of proteins being released within the first 24 h [8]. In our previous work we used pre-clotted PRP, where clots were removed prior to loading into the PEG gel (termed liquid PRP here, Figure 1). In this work, we also used liquid PRP for comparison, but added a second PRP preparation, namely powder PRP. Powder PRP was reconstituted and quickly added to the PEG microspheres during microsphere fabrication, without waiting for clots to form and then removing them (Figure 1). We posited that when clots formed inside a nanoporous inert hydrogel, they could form a secondary network (termed micro-clots here), which will sustain PRP release and impede fast hydrogel degradation. In this microsphere preparation, we were also concerned with burst release due to the significantly higher surface area for microspheres compared to a bulk PEG gel of the same volume. Burst release is typically caused by proteins which are situated at or have migrated towards the surfaces during fabrication; hence, burst release would be directly proportional to surface area. Despite the concern for burst release, encapsulation of PRP in an injectable device such as hydrogel microspheres is critical for translation to enable minimally invasive administration. As reviewed earlier, most current clinical PRP applications involve a local injection into the site of need, such as for example the knee capsule to treat osteoarthritis [11,35]. As expected, when PRP was at 10% *w*/*v* in solution (the concentration used for most of the experiments presented here), it formed large clots visible with a naked eye (Figure 1, Figure S3). Such single large clots were not observed in PEG microspheres, even though the microspheres acquired a reddish hue when loaded with PRP (Figure 5A). Note that PEG is inert, so we did not expect significant interaction between PEG and proteins within PRP. Hence, we postulate that the primary way through which PEG was influencing clot formation was confinement. 

To fabricate the microspheres, we chose to use a simple custom T-junction microfluidic setup to form hydrogel precursor solution droplets, which subsequently solidified in the collection bath (Figure 2). Droplet-based microfluidic fabrication techniques have shown great potential in offering precise control over microsphere size and size distribution [36,37]. Control of size and size distribution of hydrogel microspheres is critical in drug delivery because the rate of drug release is proportional to microsphere size. Conventional high-shear emulsion techniques such as precipitation, spray-drying, electrospraying and phase separation produce hydrogel microspheres with a large polydispersity in size and high variability in properties [28,36]. T-junction is one of the most frequently used geometries in microfluidic systems and have been used widely in the formation of droplets. Droplets of the dispersed phase are broken off by both the shear force exerted by the continuous phase (olive oil) and the squeezing effect exerted by the continuous phase when the dispersed phase (PEG) fills up the continuous phase channel [38,39]. Advantages offered by this method is that the droplet size is dependent on the channel width, the input pressure gradient, or the flow rates of the continuous and dispersed phases [40], allowing for the manipulation of multiple parameters to produce hydrogel microspheres in a variety of sizes. Note that to prevent microsphere gelation within the syringe or the microfluidic setup, microsphere fabrication was limited to ~10 min per batch, since hydrogel gelation time at pH 7.4 was ~20 min [28].

We have previously shown that by simply controlling disperse and continuous phase flow rates we can achieve microspheres in the range of ~100–500 µm (as measured directly upon fabrication and before swelling) [41]. This is a useful range, since microspheres with diameters of 50–300 µm have shown suitable for localized drug delivery via injection [42,43]. Here, all experiments were conducted with 1000 µL/min for the continuous oil phase and 10 µL/min for the disperse PEG phase, which resulted in microspheres of ~70–110 µm in diameter prior to swelling. Furthermore, our preliminary work showed that tubing diameter could also be used to manipulate microsphere size. Overall, larger tubing resulted in larger but less uniform microspheres (data not shown), hence we chose 0.5 mm (inner diameter) microfluidic tubing for all experiments. 

We noted the effect of PRP loading on microsphere size and polydispersity pre- and post-swelling (Figure 3 and Figure 4). As expected, the preparation of PRP loaded into the PEG microspheres affected their size and size distribution. When measured immediately upon fabrication, powder PRP-loaded microspheres had smaller diameters compared to both PEG-only and liquid PRP-loaded microspheres; there was no significant difference between liquid PRP-loaded and PEG-only microspheres. The decrease in the mean diameter of powder PRP-loaded microspheres could be the result of micro-clots formation during or post-gelation, providing a secondary network interpenetrating with the PEG network or simply displacing water, resulting in a smaller microsphere of lower swelling [43]. For liquid PRP, the clots that formed during PRP dissolution (prior to loading) were already removed as described in Figure 1A. 

When looking at diameters of swollen microspheres, we noted no difference between the powder PRP-loaded and PEG-only microspheres, but much larger sizes and polydispersity of liquid PRP-loaded microspheres (Figure 4). The increase in the mean diameter of swollen liquid PRP-loaded microspheres could be the result of inefficient crosslinking of the PEG network in the presence of a high concentration of soluble macromolecules [44]. Since swelling is inversely proportional to the modulus of hydrogel materials, we hypothesized that PRP loading would also affect the mechanical properties of the fabricated microspheres. Both preparations of PRP led to an increase in the microsphere modulus compared to PEG-only microspheres (Figure 5). The increase in the modulus upon powder PRP loading could be the result of PRP clotting during the microsphere fabrication process. Micro-clots would fill spaces between crosslinks, which would otherwise be filled with water or result in the formation of a secondary network, fortifying the hydrogel network. Similarly, even soluble PRP proteins at high concentration trapped in the mesh of the PEG hydrogel would lead to a higher modulus, simply by displacing water [45]. It is also possible that the higher modulus was due to interactions between the PEG polymer and certain proteins in PRP. While PEG is considered inert, our previous work has shown that certain groups and amino acids can form hydrogen bonds with the ether oxygen of PEG [46,47]. Hence, further work is needed to tease out the exact mechanism by which PRP loading contributed to increase in gel modulus. 

Another important property of a PRP delivery device is degradability, which would negate the need for removal when the payload is depleted allowing for repeatable therapy. In this study, hydrogel microspheres were fabricated using the synthetic polymer PEG. PEG has the advantages of being non-immunogenic, inert, robust, and biocompatible [26] and has been widely used in a variety of clinical applications including drug delivery [48]. PEG microspheres were formed via a step-growth Michael-type addition reaction between the acrylate group and the thiol group, resulting in a degradable thioester moiety [8]. Here we observed that powder PRP-loaded microspheres degraded much slower than PEG-only ones both in vitro (Figure 6 and Figure 8) and in vivo (Figure 7). When microspheres were prepared with liquid PRP (Figure S1), they degraded slower than PEG-only microspheres, but significantly faster than powder PRP-loaded ones. The faster degradation for the liquid PRP-loaded compared to powder PRP-loaded microspheres could be due to clots being removed before encapsulation as described earlier, implicating clotting as a major contributor to prolonged degradation upon PRP loading. Note that when other non-clotting model proteins were loaded in the PEG microspheres, the microspheres also degraded significantly faster than powder-PRP-loaded ones (Figure S2). 

We further demonstrated that micro-clotting was the reason behind the vastly different properties and degradation times for liquid vs powder PRP-loaded PEG microspheres. To do so, during the fabrication process we incorporated molecules that either prevent clotting (CaCl_2_-free PBS) or break up clots (plasmin) (Figure 9). For both Ca^2+^-free medium and plasmin conditions, we noted increased degradation at day 8 compared to the control condition (plus Ca^2+^, no plasmin), indicating that PRP micro-clots were formed inside the PEG microspheres and contributed to microsphere stability. Note that even though the microspheres were prepared in a manner to prevent or break clots, they were still soaked in PBS for the degradation studies. Hence, for all conditions, we noted that PRP released into the PBS was able to form clots outside the PEG microspheres (Figure 9). Overall, we showed that PRP micro-clotting during fabrication affected the degradation of powder PRP-loaded PEG microspheres. The PRP micro-clots inside a PEG network could be beneficial for the design of PRP delivery devices with long degradation times.

The mechanisms of how drugs are released from PEG hydrogels depend on the method of drug loading and the size and characteristics of the drug [43]. In general, diffusion and swelling-controlled delivery are well-known release mechanisms for PEG hydrogels. The effect of drug loading and size and characteristics of the drug on the release profile is also well studied [37,43,49,50]. PRP release from PEG hydrogels have been studied previously by us [8], but little is known about PRP release from PEG hydrogel microspheres and also how the PRP preparation affects the release profile. We noted lower total PRP release and slower effective protein diffusivity from powder PRP-loaded compared to liquid PRP-loaded microspheres, which could be due to micro-clotting (Figure 10). On the other hand, higher PRP total release and diffusivity from liquid PRP-loaded microspheres could be due to the higher microsphere swelling (Figure 3) compared to powder PRP-loaded microspheres. Here we also used a control DPBS release buffer, which is Ca^2+^-free and inhibits clotting during the release experiment. As expected, more total protein with a faster effective diffusivity was released for both liquid and powder PRP-loaded microspheres in DPBS compared to PBS, underscoring the role of clotting in regulating PRP release. 

While not explored here, we have previously shown that PRP remained active upon encapsulation and release from a PEG hydrogel [7,8]. PRP bioactivity for up to 14 days of release (until complete PEG gel degradation) was shown indirectly by testing the effect of PRP on primary human dermal fibroblasts [8] and primary human chondrocytes [7] proliferation. Bolus PRP was used as control in all cases. We have also previously shown certain GFs abundant in PRP, such as EGF, were continuously released until complete gel degradation, albeit with a burst release [8]. In our previous work we used the liquid PRP formulation. Future studies will focus on the differences in protein bioactivity and release profiles of specific proteins between the liquid and powder PRP formulations.

## 5. Conclusions

In this study, PRP-loaded PEG microspheres were fabricated using a custom T-junction microfluidic setup and characterized for their properties as a function of PRP loading and preparation. Two PRP preparations were used: powder PRP and liquid PRP. Loading powder PRP led to 30% decrease in mean diameter and loading of liquid PRP led to 12% increase in mean diameter compared to PEG-only microspheres. Powder PRP had no effect on swelling, while liquid PRP led to a significant 2.25-fold increase in microsphere swelling compared to PEG-only microspheres. Both powder and liquid PRP significantly increased the storage modulus of microspheres. Liquid PRP led to significantly faster degradation compared to powder PRP-loaded microspheres, but both PRP preparations suppressed degradation compared to PEG-only microspheres. PRP micro-clots in the powder PRP preparation played a role in delaying microsphere degradation as supported by the addition of plasmin and the absence of CaCl_2_ during microsphere fabrication. Sustained release of PRP was observed for both liquid and powder PRP with initial burst release. However, micro-clots present in the powder PRP slowed release of PRP compared to liquid PRP-loaded microspheres. Our study showed that we could harness the natural ability of PRP to clot, which when accomplished in a confined nanoporous PEG network, could lead to PRP micro-clotting and result in prolonged PRP release and microsphere degradation profiles. The developed microspheres could be a useful injectable PRP delivery device for the treatment of diseases such as knee osteoarthritis.

## Supplementary Materials

The following are available online at https://www.mdpi.com/2073-4360/12/8/1712/s1, Figure S1: Degradation of microspheres loaded with 10% *w*/*v* liquid PRP, Figure S2: Degradation of microspheres loaded with 10% *w*/*v* protein as a function of protein type, Figure S3: Plasmin activity.
