# Supplementary material for: Micro-Clotting of Platelet-Rich Plasma Upon Loading in Hydrogel Microspheres Leads to Prolonged Protein Release and Slower Microsphere Degradation
Source: Polymers (Basel). 2020 Jul 30;12(8):1712. doi: 10.3390/polym12081712 (PMC7464943; doi:10.3390/polym12081712)
Supplement: Supplementary file 1 [file polymers-12-01712-s001.pdf]

polymers

MDPI

Article

Micro-Clotting of Platelet-Rich Plasma Upon Loading in Hydrogel Microspheres Leads to Prolonged Protein Release and Slower Microsphere Degradation

Miran Hannah Choi [1,2], Alexandra Blanco [1,2], Samuel Stealey [1], Xin Duan [3], Natasha Case [1], Scott Allen Sell [1], Muhammad Farooq Rai [3,4] and Silviya Petrova Zustiak [1,*]

[1] Program of Biomedical Engineering, School of Engineering, Saint Louis University, 63103, Saint Louis, MO, USA. miranc2@illinois.edu (M.H.C); ablanco3@illinois.edu (A.B); samuel.stealey@slu.edu (S.S); natasha.case@slu.edu (N.C); scott.sell@slu.edu (S.A.S)

[2] Current address: Department of Bioengineering, University of Illinois at Urbana-Champaign, 61801, Urbana, IL, USA

[3] Department of Orthopedic Surgery, Washington University in St. Louis, School of Medicine, 63110, Saint Louis, MO, USA. duan.x@wustl.edu (X.D); rai.m@wustl.edu (M.F.R)

[4] Department of Cell Biology & Physiology, Washington University in St. Louis, School of Medicine, 63110, Saint Louis, MO, USA

* Correspondence: silviya.zustiak@slu.edu; 314-977-8331

Received: 30 June 2020; Accepted: 28 July 2020; Published: dateSupplemental Information

Supplemental Information

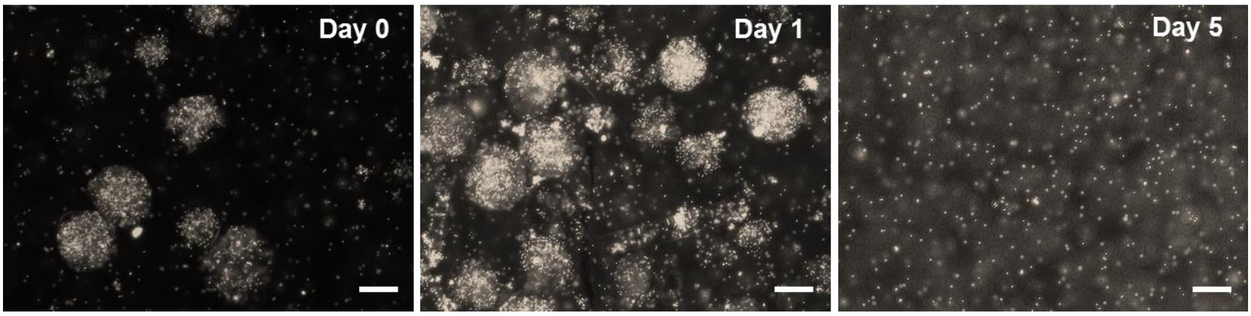

Figure 1. **Degradation of microspheres loaded with 10% w/v liquid PRP.** Microspheres were prepared with four-arm PEG-Ac and PEGDD-1 as a crosslinker at 7.5% w/v in PEG. Red fluorescent beads were added to the microspheres for visualization. The scale bar is 100 μm.

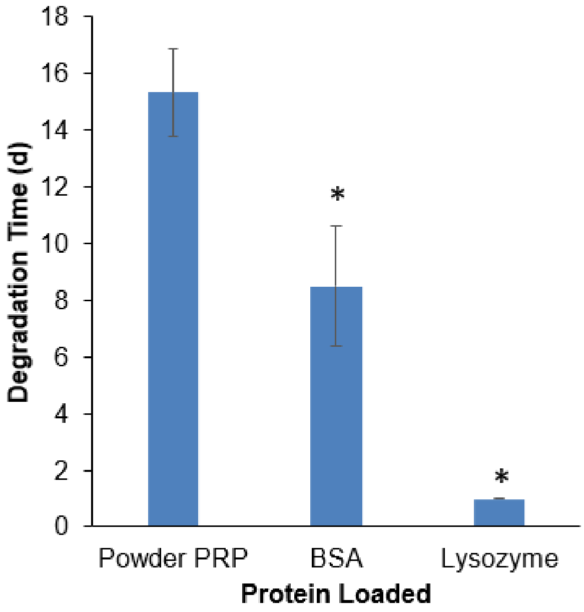

Figure 2. **Degradation of microspheres loaded with 10% w/v protein as a funciton of protein type.** Microspheres were prepared with four-arm PEG-Ac and PEGDD-1 as a crosslinker at 7.5% w/v in PEG and loaded with powder PRP, BSA or lysozyme. *denotes significant difference from powder PRP ($n = 50$; $p < 0.05$)..

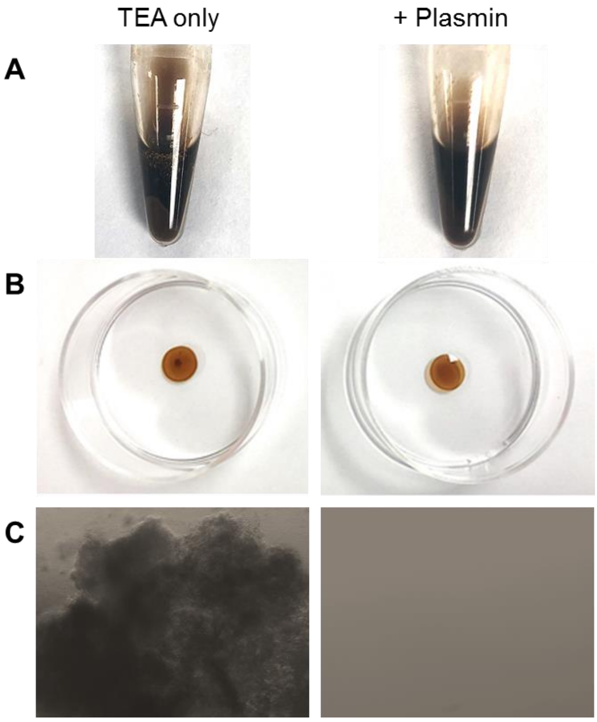

Figure 3. **Plasmin activity.** (**A, B**) Macroscopic images of powder PRP at 10% w/v reconstituted in TEA buffer only or in TEA buffer containing 1% v/v plasmin. (**B)** Microscopic images of powder PRP at 10% w/v reconstituted in TEA buffer only or in TEA buffer containing 1% v/v plasmin.
